# Risk of Type 2 Diabetes Mellitus following Gestational Diabetes Pregnancy in Women with Polycystic Ovary Syndrome

**DOI:** 10.1155/2017/5250162

**Published:** 2017-12-20

**Authors:** Joan C. Lo, Jingrong Yang, Erica P. Gunderson, Mohammad K. Hararah, Joel R. Gonzalez, Assiamira Ferrara

**Affiliations:** Division of Research, Kaiser Permanente Northern California, Oakland, CA, USA

## Abstract

**Background:**

This study examines gestational diabetes mellitus (GDM) in women with polycystic ovary syndrome (PCOS) and the risk of type 2 diabetes mellitus (DM) following GDM pregnancy.

**Methods:**

A cohort of 988 pregnant women with PCOS who delivered during 2002–2005 was examined to determine the prevalence and predictors of GDM, with follow-up through 2010 among those with GDM to estimate the risk of DM.

**Results:**

Of the 988 pregnant women with PCOS, 192 (19%) developed GDM. Multivariable predictors of GDM included older age, Asian race, prepregnancy obesity, family history of DM, preconception metformin use, and multiple gestation. Among women with PCOS and GDM pregnancy, the incidence of DM was 2.8 (95% confidence interval (CI) 1.9–4.2) per 100 person-years and substantially higher for those who received pharmacologic treatment for GDM (6.6 versus 1.5 per 100 person-years, *p* < 0.01). The multivariable adjusted risk of DM was fourfold higher in women who received pharmacologic treatment for GDM (adjusted hazard ratio 4.1, 95% CI 1.8–9.6). The five-year incidence of DM was 13.1% overall and also higher in the pharmacologic treatment subgroup (27.0% versus 7.1%, *p* < 0.01).

**Conclusions:**

The strongest predictors of GDM among women with PCOS included Asian race and prepregnancy obesity. Pharmacologic treatment of GDM is associated with fourfold higher risk of subsequent DM.

## 1. Introduction

Women with polycystic ovary syndrome (PCOS) are at increased risk for developing gestational diabetes mellitus (GDM) during pregnancy [1–6]. Both PCOS and GDM are also risk factors for type 2 diabetes mellitus [1, 2, 7–9]. Women with GDM pregnancy have a 7-fold higher risk of developing subsequent type 2 diabetes mellitus compared to women without GDM [10], with an annual incidence of 1.7–2.2% [11–13]. In women with PCOS, the risk of diabetes has been reported up to 8-fold higher compared to that in women without PCOS [7]. While fewer studies have examined diabetes risk in women with PCOS, women who experience a GDM pregnancy, impaired glucose metabolism, and glucose intolerance following GDM pregnancy has been observed [14, 15]. In this study, we examined a large, community-based population of women with PCOS and GDM pregnancy to determine the subsequent risk of diabetes and differential risk by GDM severity.

## 2. Materials and Methods

Kaiser Permanente Northern California (KPNC) is an integrated healthcare delivery system with over 3 million members and more than 30,000 births/year. We identified nondiabetic KPNC women with PCOS who had a delivered pregnancy during 1 January 2002 to 31 December 2005 and underwent screening for GDM during pregnancy. Women identified with GDM were followed after pregnancy delivery to 31 December 2010 for the development of type 2 diabetes mellitus, censoring at diabetes, membership disenrollment, death, or end of follow-up (whichever came first). The study was approved by the KPNC Institutional Review Board, and the requirement for informed consent was waived due to the nature of the study.

As previously described [16, 17], chart review was conducted to verify PCOS diagnosis using the 2003 ESHRE/ASRM Rotterdam criteria [9], requiring at least two of the three criteria: oligo- or amenorrhea, androgen excess, and polycystic ovary morphology by ultrasound. Androgen excess was defined by acne, hirsutism, and/or elevated androgen levels. Polycystic ovary morphology was verified by imaging, except for 5% of the cohort where records were unavailable to verify reproductive endocrine documentation of polycystic-appearing ovaries [16]. Patient characteristics, including preconception body mass index (BMI), metformin, fertility drugs or *in vitro fertilization* [16, 17], family history of diabetes, and pharmacologic management of GDM, were ascertained by chart review and pharmacy records.

The diagnosis of GDM was based on a 3 hr 100 g oral glucose tolerance test (OGTT) using the American Diabetes Association criteria during the study period [8]. We additionally included women with 1 hr 50 g screening glucose ≥ 180 mg/dL (10 mmol/L) who were managed as GDM patients without a 3-hr OGTT (*N* = 18). Subsequent development of type 2 diabetes was established by laboratory evidence of fasting glucose ≥ 126 mg/dL (7.0 mmol/L), HbA1C ≥ 6.5%, or treatment with a hypoglycemic agent (insulin or glyburide), except during a subsequent GDM pregnancy. Metformin treatment in the absence of laboratory criteria did not qualify for diabetes, given its known use in PCOS management. When both fasting glucose and HbA1C criteria were met, the date of diabetes was determined by fasting glucose criteria.

### 2.1. Statistical Methods

Differences between subgroups were compared using chi-square, Fisher exact or Student's *t*-tests. Multivariable logistic regression was used to examine independent predictors of GDM. For women with PCOS who experienced a GDM pregnancy, the subsequent incidence of diabetes was calculated per 100 person-years. The association of GDM pharmacotherapy and risk of subsequent diabetes mellitus was examined using Cox proportional hazard analyses, adjusted for potential confounders. Analyses were performed using SAS version 9.3 (SAS Institute, Cary, NC) or STATA version 10.2 (College Station, TX); a two-sided *p* value of <0.05 was chosen as the criterion for statistical significance.

## 3. Results

Among 1023 women with PCOS and delivered pregnancy, 988 (96.6%) underwent GDM screening. The remaining 35 women not screened for GDM included 17 who delivered extremely preterm (≤29 weeks of gestation) and 11 who were managed presumptively as GDM patients. Among the 988 women screened for GDM, 174 (17.6%) met the GDM criteria based on the 3-hour OGTT [8] and 18 with 1-hour glucose ≥ 180 mg/dL (10.0 mmol/L) following 50 g oral glucose were managed as GDM patients without a 3-hour OGTT, yielding a total of 192 (19.4%) pregnant women with PCOS and GDM. Within this subset, 57 (29.7%) received pharmacologic treatment for GDM (insulin or glyburide).

As previously described [17], the cohort was racially/ethnically diverse, with 41.0% White, 25.5% Asian, 26.0% Hispanic, 3.9% Black, and 3.6% other race. The mean age was 31.4 ± 4.4 years, and 42.4% were obese. As expected, women with PCOS who had GDM were older and more likely to be Asian compared to those without GDM (Table 1). They were also more likely to be obese, have a family history of diabetes, receive preconception metformin, undergo fertility treatment, and experience multiple gestation. In multivariable logistic regression analyses adjusted for these clinical factors, predictors of GDM included older age, Asian race, family history of diabetes, moderate and severe prepregnancy obesity, preconception metformin use, and multiple gestation, with severe obesity and Asian race as the strongest risk factors (Table 1). Although treatment with fertility drugs or in vitro fertilization for conception may be markers for PCOS severity (and hence metabolic perturbation), fertility treatment was no longer associated with risk of GDM in adjusted analyses.

Among the 192 women with PCOS and GDM pregnancy, 191 (99.5%) remained health plan members following delivery. There were five incident cases of type 2 diabetes within the first year, followed by 25 cases during follow-up. Because these five cases may represent preexisting undiagnosed diabetes, the remaining analyses focus on the 25 incident cases beyond the first year among 186 PCOS GDM women (total follow-up 951.9 person-years). Those who developed diabetes had significantly higher BMI and were much more likely to have received pharmacologic therapy for GDM, whereas differences in preconception metformin use and family history of diabetes were not significant (Table 2).

**Figure 1:**
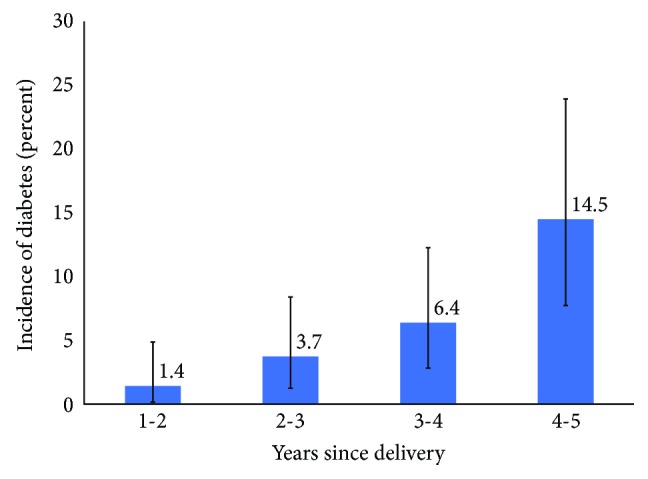
Incidence of diabetes mellitus by year of follow-up after pregnancy delivery among women with PCOS and gestational diabetes mellitus.

Figure 1 shows the incidence of diabetes per year of follow-up for the first five years after pregnancy delivery. The overall incidence of diabetes was 2.8 (CI 1.9–4.2) per 100 person-years during follow-up through 2010. The overall incidence was more than 4-fold higher if pharmacologic GDM treatment was required (6.6 versus 1.5 per 100 person-years, *p* < 0.01) compared to no pharmacologic treatment for GDM. Among the 122 women with PCOS and GDM who had complete 5-year follow-up postdelivery, the cumulative 5-year risk of diabetes was 13.1% overall and 27.0% versus 7.1% (*p* < 0.01) for those with and without pharmacologic GDM treatment, respectively. Adjusted for age, race/ethnicity, prepregnancy BMI, family history of diabetes, and preconception metformin use, pharmacologic treatment for GDM was associated with a 4-fold higher rate of incident diabetes (hazard ratio 4.1, CI 1.8–9.6; Table 2).

## 4. Discussion

Among nearly 1000 women with Rotterdam diagnosis-confirmed PCOS, we found a high prevalence of GDM at 19%, 2-3 times higher than the KPNC background rate of 6%–8% [18]. Our prevalence of GDM among women with PCOS is higher than previously estimated at 14% when PCOS was identified solely by coded diagnosis [3], without chart review for greater cohort specificity. We found that women with PCOS who were of Asian race had significantly higher risk of GDM, consistent with previous observations for the general population [18]. For women with PCOS who experienced a GDM pregnancy, the subsequent incidence of diabetes was 2.8 per 100 person-years overall and fourfold higher if pharmacologic treatment for GDM was required. In other GDM cohorts not selected for PCOS, insulin therapy has been associated with 3–5-fold higher risk of postdelivery diabetes compared to no insulin therapy [19–21].

It is estimated that 10%–50% of women with GDM develop diabetes during the 5-year interval following delivery [12, 22]. A prospective study that excluded preexisting diabetes based on prepregnancy glycemia measurements reported a diabetes incidence rate of 1.8 per 100 person-years among GDM women assessed on average within ten years after their last pregnancy [11]. Diabetes incidence rates of 1.7–2.2 per 100 person-years have also been reported in other GDM populations not selected for PCOS status [12, 13]. Our data suggest the risk of diabetes among PCOS women with prior GDM is much higher. While racial/ethnic differences in diabetes risk were not observed in our study, the numbers within each non-White subgroup were limited. Others have found differential diabetes risk conferred by GDM pregnancy for White compared to Asian women [23] and higher risk among African American women [24], not considering PCOS status. While Asian race is a predictor of GDM in women with PCOS [5], among the broader population of women with GDM irrespective of PCOS status, Asian race is also a predictor of subsequent type 2 diabetes [12].

Our study has some limitations. Although postpartum diabetes surveillance is likely greater for PCOS women, the variable rate of diabetes screening in routine clinical care during the period of our study (including the use of fasting glucose and/or hemoglobin A1C rather than that of an oral glucose tolerance test) may contribute to potential underestimation of diabetes incidence. Furthermore, women diagnosed with diabetes in their first postpartum year were not included. We also did not examine relevant postpartum factors such as weight trajectory (or postpartum weight retention), physical activity, lactation, or subsequent reproductive care (including metformin) that may have influenced diabetes risk. Nonetheless, this is one of the first studies from a large US healthcare delivery system examining GDM and subsequent diabetes risk in women with chart-confirmed PCOS, where receipt of pharmacologic GDM therapy was associated with a 4-fold higher risk of subsequent diabetes. Collectively, these findings emphasize the importance of systematic diabetes screening in this high-risk population [1] and further studies examining racial/ethnic differences in the outcome, the role of androgen excess, weight status, and modifiable risk factors.

## Figures and Tables

**Table 1:** Baseline characteristics in pregnant women with polycystic ovary syndrome (PCOS) by gestational diabetes mellitus (GDM) status.

		988 PCOS women by GDM status			Adjusted odds of GDM	
		No GDM *N* = 796	GDM *N* = 192	*p* value	Adjusted^∗^ odds ratio	95% confidence interval
Age in years (mean ± standard deviation)		31.1 ± 4.5	32.9 ± 4.0	<0.01	1.11	1.06–1.15
Race/ethnicity						
White		43.5%	30.7%	<0.01	Referent	
Black		4.0%	3.1%		0.95	0.36–2.49
Hispanic		26.1%	25.5%		1.42	0.91–2.21
Asian		22.5%	38.0%		3.52	2.26–5.47
Other		3.9%	2.6%		0.89	0.31–2.51
Prepregnancy BMI						
Normal	<25.0 kg/m^2^	30.7%	19.3%	<0.01	Referent	
Overweight	25–29.9 kg/m^2^	30.0%	25.5%		1.42	0.86–2.33
Moderately obese	30–39.9 kg/m^2^	30.2%	39.1%		2.65	1.63–4.32
Severely obese	≥40.0 kg/m^2^	9.2%	16.2%		4.04	2.16–7.57
Family history of diabetes		54.7%	64.6%	0.01	1.52	1.07–2.17
Preconception metformin^†^		21.6%	35.9%	<0.01	1.65	1.14–2.39
Treatment with fertility drugs or IVF^‡^		56.7%	66.7%	0.01	1.19	0.82–1.73
Multiple gestation pregnancy		9.7%	16.7%	<0.01	1.88	1.14–3.10

^∗^Covariates in the adjusted logistic regression model. ^†^Administered within 3 months of conception to assist in achieving ovulatory cycles. ^‡^In vitro fertilization.

**Table 2:** Baseline characteristics and incident diabetes mellitus (DM) in women with polycystic ovary syndrome (PCOS) and gestational diabetes mellitus (GDM) pregnancy.

	186^∗^ PCOS women with GDM by subsequent DM status			Adjusted relative rate of DM following GDM pregnancy	
	No diabetes *N* = 161	Diabetes *N* = 25	*p* value	Adjusted hazard ratio	95% confidence interval
Age, years (mean ± standard deviation)	32.9 ± 4.1	33.3 ± 3.4	0.57	1.01	0.91–1.12
Race/ethnicity					
White	31.1%	32.0%	0.44	Referent	
Black	3.1%	0.0%		—^§^	
Hispanic	23.0%	40.0%		1.38^§^	0.51–3.74
Asian	39.8%	28.0%		1.96	0.55–6.94
Other	3.1%	0.0%		—^§^	
Prepregnancy BMI (kg/m^2^)	31.6 ± 7.6	37.5 ± 7.1	<0.01	1.09	1.03–1.15
Family history of diabetes	62.7%	80.0%	0.12	1.59	0.56–4.46
Preconception metformin^†^	34.2%	48.0%	0.18	0.91	0.39–2.11
Pharmacologic treatment for GDM	23.6%	60.0%	<0.01	4.10	1.75–9.57

^∗^One woman with no follow-up after delivery and 5 women with incident DM during the first year after delivery were not included. ^†^Administered within 3 months of conception to assist in achieving ovulation. ^§^Women of other (*N* = 5) or Black (*N* = 5) race were included with the 47 women of Hispanic ethnicity for these analyses.
